# Compression therapy following ClariVein® ablation therapy: a randomised controlled trial of COMpression Therapy Following MechanO-Chemical Ablation (COMMOCA)

**DOI:** 10.1186/s13063-019-3787-4

**Published:** 2019-12-05

**Authors:** Doireann P. Joyce, Stewart R. Walsh, Charyl J. Q. Yap, Tze T. Chong, Tjun Y. Tang

**Affiliations:** 1 Department of Vascular Surgery, Galway University Hospital, Galway, Ireland; 2 Department of Vascular Surgery, Singapore General Hospital, Singapore, Singapore

**Keywords:** ClariVein®, Mechano-chemical ablation, Varicose veins, Truncal ablation, Compression

## Abstract

**Background:**

Endovenous treatment of varicose veins has increased in popularity over the last decade. There remains, however, a degree of uncertainty regarding the role of compression bandaging or hosiery following this intervention. The National Institute for Clinical Excellence Guideline Development Group has advocated further research to evaluate the clinical and cost-effectiveness of this post-procedure intervention. In addition to this, the duration of compression bandaging also warrants clarification.

**Methods:**

Ethical approval for the study was obtained from the Singhealth Centralised Institutional Review Board (CIRB Ref: 2017/2710). Consent to enter the study will be sought from each participant only after a full explanation has been given, an information leaflet offered and time allowed for consideration. Signed participant consent will be obtained. Patients will be randomised to either compression (group A) or no compression (group B). The primary aim of the study is to assess the patient’s pain scores for the first 10 days post procedure using a visual analogue scale. Secondary aims include an assessment of patient compliance with compression, quality of life scores, clinical effectiveness, rates of bruising and phlebitis, time taken to return to normal activities, patient satisfaction and occlusion rate at 6 months.

**Discussion:**

The purpose of this study is to examine the effect of compression therapy in patients having mechano-chemical ablation (MOCA) therapy for truncal incompetence of their varicose veins using the ClariVein® device. This study may provide clarification on the role of compression therapy in patients undergoing MOCA.

**Trial registration:**

ClinicalTrials.gov, NCT03685838. Registered on 26 September 2018.

## Background

Varicose veins are common and are known to affect approximately one-third of the population [1]. Chronic venous disease (CVD) has been shown to have a negative impact on the quality of life of patients and treatment of varicose veins has been demonstrated to lead to improvement in quality of life [2–4]. Over the past decade, new endovenous techniques have been introduced and these are felt to be cost-effective, especially when performed in an outpatient or ‘office-based’ setting [5]. There is currently uncertainty about the use of compression stockings following treatment of varicose veins. In their consensus statement in 2008, the International Union of Phlebology (IUP) stated that there is good evidence for using compression in certain clinical indications [6]. These include the management of telangiectasia after sclerotherapy, varicose veins in pregnancy, prevention of thromboembolism and healing of ulcers. However, a few questions remain unanswered, such as the length of treatment and level of compression to be used [6]. The Society for Vascular Surgery and the American Venous Forum recommend using compression stockings post-operatively for 1 week to prevent haematoma formation, pain and swelling [7]. The 2013 NICE Guidelines on Varicose Veins in the Legs recommended that compression hosiery is used for no more than 7 days after interventional treatment for varicose veins [8]. However, due to current uncertainty of compression bandaging or hosiery compared to no compression after interventional treatment for varicose veins, the NICE Guideline Development Group has advocated further research to evaluate the clinical and cost effectiveness of this post-procedure intervention [8]. The guidelines also suggested looking into the length of time compression bandaging should be worn if it is shown to be beneficial [8].

Several researchers have looked into the practice of using compression after venous ablation. In a survey of the management of varicose veins by the members of the Vascular Society of Great Britain and Ireland, Edwards et al. [9] found that the majority of surgeons used bandages post-operatively, with 49% using elastic bandages. The literature on the use of compression stockings following treatment of varicose vein is limited.

Between December 2006 and February 2008, Bakker et al. [10] conducted a prospective randomised controlled trial on the use of compression stockings after endovenous laser ablation of the great saphenous vein. One hundred and nine patients were approached, with 93 finally randomised to use compression stockings for 2 days (Group A) and 7 days (Group B), respectively. All patients were followed up for 3 months post treatment and the visual analogue scale (VAS) for pain was recorded at 48 h, 1 week and 6 weeks. A physical examination and quality of life were assessed at 1 week and 6 weeks. The occlusion rate at the 3-month point was also evaluated. Physical function and vitality were shown to be significantly better in group B at 1 week follow-up, but there was no statistically significant difference at 6 weeks. At 1 week, it was also noted that the VAS score in group B (VAS score 2.0 ± 1.1) was significantly lower than in patients wearing compression stockings for 48 h (VAS score 3.7 ± 2.1) (*p* ≤ 0.001) [10]. No significant difference, however, was observed at 6 weeks post procedure. Limitations of the study include the high drop-out of the trial (40 out of the initial 109 approached) and the absence of any phlebectomies or sclerotherapy in the patients.

Elderman et al. [11] carried out a randomised trial to assess the effect of compression stockings after endovenous laser therapy (EVLT) for great saphenous vein incompetence. Patients’ reported pain scores and quality of life scores were evaluated on the day of the procedure, 2–3 days afterwards and 2–6 weeks post procedure. A total of 111 patients were randomised to stockings (*n* = 55) and no stockings (*n* = 56). There was a statistically significant difference in the pain scores in favour of the stockings group up to day 7, but this difference was no longer present by week 6. There was also a greater use of analgesia in patients in the no stockings group compared to patients wearing stockings (*p* < 0.05). In addition, patients wearing stockings reported a statistically significantly higher score of satisfaction at 2 days (4.44 vs 4.15) and at 6 weeks (4.59 vs 4.18). The absolute difference, however, was small. Two notable limitations of the study were the high level of dropouts (16 from each group) and the absence of any blinding.

Hamel-Desnos et al. [12] undertook a randomised controlled trial looking at the effect of compression in patients receiving foam sclerotherapy of the saphenous vein. They noted that patients with compression had similar pain and quality of life scores to patients not wearing any compression. They concluded that additional use of compression had no impact on the effectiveness of obliteration of veins, satisfaction scores, symptoms and quality of life, and that further controlled trials were needed to answer the question of whether using compression results in any difference to the outcome of varicose vein procedures. A summary of the available evidence is presented in Table 1.

**Table 1:** Summary of published studies related to the COMMOCA trial

Author	Study arms	Number of patients (*n*)	Timeframe	Outcomes	Limitations
Bakker et al. [10]	Compression × 2 days (Group A) vs compression × 5 days (Group B)	93 patients randomised to stockings (*n* = 48) and no stockings (*n* = 45)	Pain scores recorded using VAS at 48 h, 1 week and 6 weeks. Total follow-up of 3 months	Physical function and vitality significantly better in group B at 1 week. No statistically significant difference at 6 weeks. VAS score in group B (2.0 ± 1.1) was significantly lower at 1 week than in group A (3.7 ± 2.1, *p* ≤ 0.001) [10]. No significant difference, observed at 6 weeks post procedure	High drop-out of the trial Absence of any phlebectomies or sclerotherapy
Elderman et al. [11]	Stockings vs no stockings	111 patients randomised to stockings (*n* = 55) and no stockings (*n* = 56)	Pain scores and quality of life scores evaluated on the day of the procedure, 2–3 days afterwards and 2–6 weeks post procedure	Statistically significant difference in the pain scores in favour of the stockings group up to day 7. No significant difference at 6 weeks Greater use of analgesia in patients in the no stockings group (*p* < 0.05). Significantly higher satisfaction scores in stockings group at 2 days (4.44 vs 4.15) and at 6 weeks (4.59 vs 4.18)	High level of dropouts and the absence of blinding
Hamel-Desnos et al. [12]	Compression vs no compression	60 patients randomised to compression (*n* = 31) vs no compression (*n* = 29)	Clinical and duplex ultrasound assessments made on days 14 and 28 post procedure	No difference between compression and control groups when comparing efficacy, side effects, satisfaction scores, symptoms and QOL	Poor compliance with compression therapy

*COMMOCA* COMpression Therapy Following MechanO-Chemical Ablation, *QOL* quality of life; *VAS* visual analogue scale

Mechano-chemical ablation (MOCA) combines mechanical damage to the endothelium caused by a rotating wire with simultaneous catheter-guided infusion of a liquid sclerosant that irreversibly damages the cellular membranes of the endothelium, causing fibrosis of the vein. The exact mechanism is still not exactly known; however, recent experimental research showed that various sclerosants induced apoptosis in the vein wall rather than having an effect restricted to the endothelium. Incomplete loss of endothelial cells and penetration of the sclerosant effect into the media suggest that medial damage is crucial to the success of sclerotherapy and may explain why it is less effective in larger veins [13]. This poses the question of whether one needs compression post sclerotherapy to improve contact of the sclerosant to the endothelium when media penetration seems to be more important to allow apoptosis of smooth muscle cells.

We, therefore, propose to undertake a randomised study looking at the effect of compression therapy after mechano-chemical ablation using the ClariVein® (www.clarivein.com) device.

## Methods

### Aims

The primary aim of the study is to assess patient’s pain scores for the first 10 days post procedure using a visual analogue scale (VAS). Secondary aims are to compare the two treatment groups with respect to: quality of life scores at baseline, 2 weeks and 6 months using the EQ-5D (EuroQol 5 Dimensions), AVVQ (Aberdeen Varicose Vein Questionnaire) and CIVIQ (ChronIc Venous Insufficiency quality of life Questionnaire) scores; clinical change using the VCSS (Venous Clinical Severity Score) at baseline, 2 weeks and 6 months; the degree of bruising (mild, moderate and severe) and phlebitis at 2 weeks and 6 months; patient compliance with the intervention; time taken to return to work and normal activities; patient satisfaction, assessed with a patient satisfaction questionnaire; and successful obliteration of the target vein, assessed with duplex ultrasound scan at 6 weeks and 6 months. Recanalisation will be defined by a segment of vein ≥ 5 cm. A comparison of the cost-effectiveness of the intervention will also be carried out.

### Study design

This will be a prospective, multi-centre, international randomised controlled trial.

### Study setting

The trial will take place in Singapore General Hospital, Singapore and Roscommon University Hospital, Ireland.

### Target population

Patients referred for treatment of symptomatic varicose veins or chronic venous insufficiency will be recruited if they are found to have primary great saphenous vein (GSV) or small saphenous vein (SSV) incompetence on colour Duplex scan.

### Enrolment criteria

Inclusion criteria for this study include: age ≥ 18 to ≤ 80 years; ability to walk unassisted and ability to attend follow-up visits; and symptomatic GSV or SSV vein reflux > 0.5 s on colour Duplex ultrasound.

Exclusion criteria are as follows: previous or current deep vein thrombosis or pulmonary embolism; patients with a hypercoagulable state; patients on warfarin or novel oral anticoagulants; patients with previous thrombophlebitis in the truncal vein in question, which had recanalised and was now incompetent on duplex ultrasound; recurrent varicose veins (i.e. patients who have had treatment previously in the designated truncal vein with any modality); patients who have had treatment in either leg for an incompetent saphenous truncal vein less than 3 months prior to treatment and enrolment into this study; patients requiring adjuvant treatment of varicose veins; arterial disease (Ankle–Brachial Pressure Index (ABPI) < 0.6 and the absence of a palpable pedal pulse); vein diameter < 3 mm or > 12 mm [14] as measured in the standing position on duplex ultrasound; patients who are unwilling to participate; inability or unwillingness to complete questionnaires; patients unable to provide informed consent or comply with the study protocol; varicose veins unsuitable for MOCA (e.g. very tortuous veins); pregnancy; lycra™ (a type of elastic fabric and fibre used for tight-fitting garments), sclerosant or local anaesthetic allergy; patients who have opted for an alternative method of treatment; patients with a life expectancy less than 12 months; patients with fibromyalgia; patients on anticoagulation with warfarin; and patients with CEAP (Clinical–Etiological–Anatomical–Pathophysiology) Score of C6 (active ulcer) or C1 and C2 (asymptomatic) disease.

### Description of intervention

Patients will be randomised (using computer-generated random numbers in sequentially numbered, opaque, sealed envelopes) to have compression (group A) or no compression (group B). The compression therapy used will be Class II (18–24 mmHg) above-knee compression stockings. Patients randomised to group A will be asked to wear compression stockings for 1 week. This would involve wearing the stocking during the daytime but patients will be allowed to take it off at night whilst in bed. Patients randomised to group B will not be provided with any compression. Those requiring concurrent phlebectomies will be excluded. Patients allocated to compression will all wear the same length, style and grade of stockings.

### Baseline

At baseline, patients will be asked to fill quality of life questionnaires (EQ-5D, AVVQ and CIVIQ; Additional files 1, 2 and 3) and will have their clinical scores assessed (CEAP and VCSS; Additional files 4 and 5). On discharge after their varicose vein intervention, the patients will then be provided with a diary to record their post-procedural pain every day for 10 days using a validated visual analogue scale (VAS) as well as to record when they return to their normal activities and are back to work (Additional files 6 and 7, respectively). They will also be asked to attend a follow-up in 2 weeks and at 6 months.

### ClariVein® technique

Patients will be consented for ClariVein®, being a relatively new technique under study. All patients will receive a procedure-specific information leaflet in their native language, which explains the technique including risks and side effects as well as a description of alternative techniques. Patients who do not want to be treated with ClariVein® will be routinely offered treatment with RFA (radiofrequency ablation), VenaSeal™ or open surgery.

In keeping with local preference, patients will be offered the procedure under local anaesthetic (lidocaine 1%) ± sedation. Antibiotics at induction will be routinely given. The patient is positioned supine with a sandbag under the knee to enhance access to the GSV. The SSV can be treated with the patient placed either prone or in the lateral position depending on the surgeon’s preference. A Seldinger technique is used to introduce a micro-catheter 4-F or 5-F introducer sheath into either the great saphenous vein (GSV) or the short saphenous vein (SSV) under ultrasound guidance and flushed with saline. The ClariVein® infusion catheter tip is inserted through the sheath and the tip of the dispersion wire is positioned 20 mm distal to the SFJ, or for the SSV just proximal to the fascial curve. Wire rotation is activated for a 3-s period to induce spasm of the proximal vein prior to commencing pullback. With the wire continuing to rotate, infusion of the sclerosant is started simultaneously with catheter pullback. The activated catheter, which is connected to a 9-V battery-motorised handle, is steadily withdrawn at 1 cm every 7–8 s. The sclerosant used will be 2.0% liquid sodium tetradecyl sulphate (STS) for both the GSV and the SSV. The sclerosant volume used will be 0.1–0.2 ml every 1-cm pullback and will be determined by the vein diameter and overall treatment length. A completion Duplex ultrasound will be performed after the procedure to confirm the patency of the common femoral vein and the deep venous system, and to ascertain whether there is any flow within the truncal vein and whether it is still compressible. The ipsilateral foot will be dorsi-flexed and plantar-flexed in order to minimise deep venous stasis at the end of the procedure [15]. Subcutaneous heparin is routinely given after the procedure to minimise the risk of deep vein thrombosis. A full-length compression bandage (Class II; Coban lite™) will be applied to the treated limb(s) from the foot to the groin. The patient will then be advised to undertake light exercise (3 × 15-min walks on the same day) when they felt well enough to do so. Bandages will be removed in 24 h and patients will be advised to wear compression stockings for 1 week during the daytime if the patient is randomised to the compression arm of the study. Otherwise, the patient will be discharged without compression hosiery.

### Follow-up

Patients will be followed up in the outpatient clinic at 2 weeks and 6 months. At the 2 weeks’ follow-up, the diary containing details of the pain scores and how soon patients were able to return to normal activities/work will be collected. In addition, patients will be asked about any bruising or phlebitis they have had in the 2 weeks after their procedure and how compliant they have been with the compression. The degree of bruising and phlebitis will be assessed using a pre-determined score (0, 0%; 1, < 25% of treated vein affected; 2, 25–50% of treated vein affected; 3, 50–75% of treated vein affected; 4, 75–100% of treated vein affected; and 5, extending beyond the treated vein). They will be examined and the Venous Clinical Severity Score (VCSS) will be recorded. They will also be asked to fill in the EQ-5D, AVVQ and CIVIQ scores. They will have a venous Duplex scan to determine occlusion of the treated vein. Recanalisation will be defined by a segment ≥ 5 cm.

**Fig. 1:**
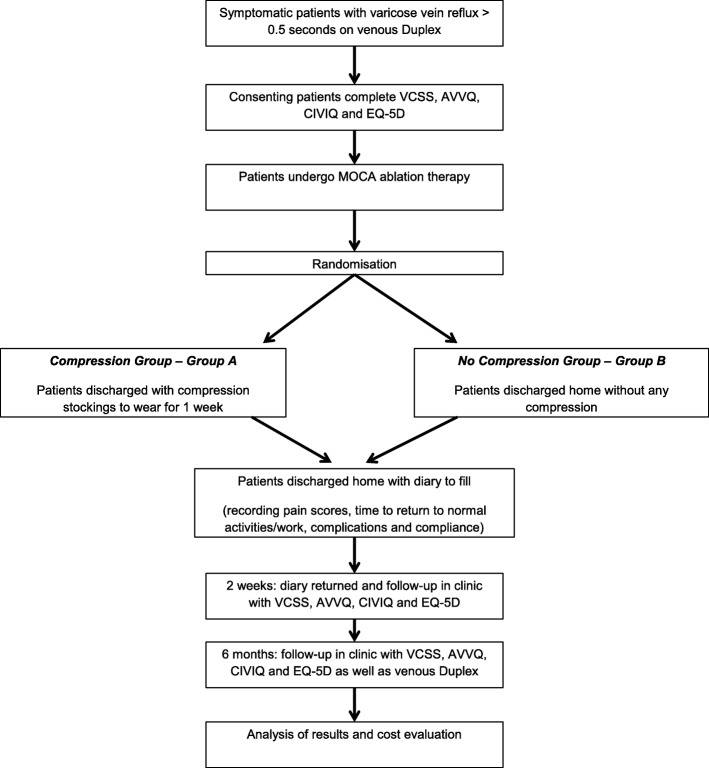
COMpression Therapy Following MechanO-Chemical Ablation (COMMOCA) randomised controlled trial flow chart. AVVQ Aberdeen Varicose Vein Questionnaire, CIVIQ ChronIc Venous Insufficiency quality of life Questionnaire, EQ-5D EuroQol 5 Dimensions, MOCA mechano-chemical ablation, VCSS Venous Clinical Severity Score

**Fig. 2:**
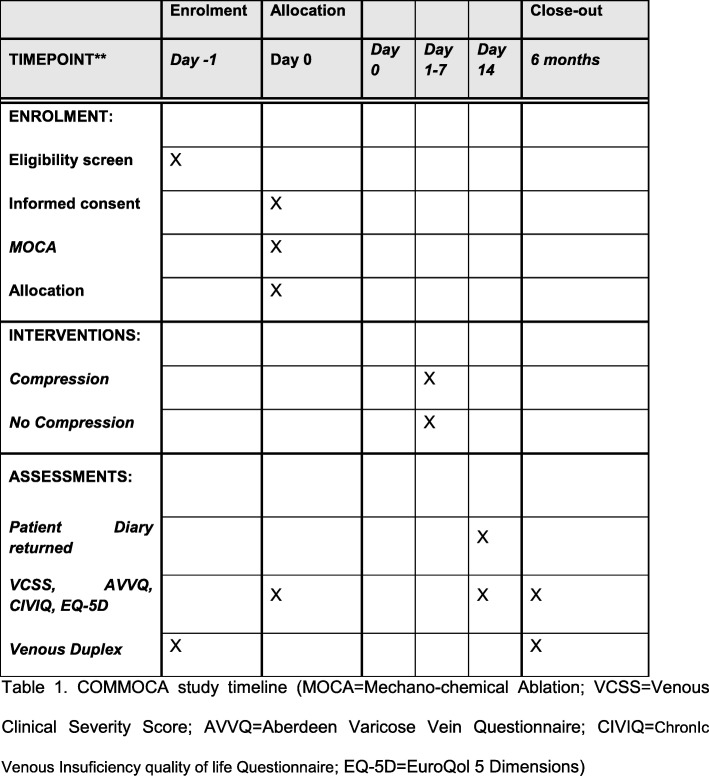
Standard Protocol Items: Recommendations for Interventional Trials (SPIRIT) flow sheet schedule of enrolment, interventions and assessments. AVVQ Aberdeen Varicose Vein Questionnaire, CIVIQ ChronIc Venous Insufficiency quality of life Questionnaire, EQ-5D EuroQol 5 Dimensions, MOCA mechano-chemical ablation, VCSS Venous Clinical Severity Score

At the 6-month follow-up, patients will be examined and their VCSS will be recorded. They will also be asked to fill in the EQ-5D, AVVQ and the CIVIQ scores. They will have a venous Duplex scan to determine occlusion of the treated vein. An overall satisfaction survey will also be asked. A flowchart for the trial is provided in Fig. 1. The study timeline is also detailed in the SPIRIT (Standard Protocol Items: Recommendations for Interval Trials) schedule of enrolment, interventions and assessments in Fig. 2. The SPIRIT checklist is included in Additional file 8.

### Sample size and study duration

The sample size needed to observe a difference of at least 20 mm in the VAS score with a standard deviation of 20 mm was estimated. With power of 90% and 5% significance equivalence, a minimum of 94 patients (47 per group) will need to be recruited. Accounting for losses to follow-up in the order of 10% gives a target sample size of 103 patients.

Previous studies looking at compression stockings have shown high drop-out rates close to 40% at 3 months. We do not anticipate this problem with the set up in the outpatient clinic at Changi General Hospital in Singapore. If we estimate 10% drop-out, this would mean we would need near to 200 patients to be included in this study. If three patients are recruited per week, this would take approximately 17 months to recruit the necessary number of patients. With 6 months follow-up, therefore, the study will be running for 23 months, but 3 years would give a good safety net for recruitment and follow-up, and allow for dropouts. We aim to have recruited 100 patients within the first 12 months of the study.

### Statistical analysis

All analysis will be undertaken on an intention-to-treat basis. Pain score data will be treated as non-parametric and compared using the Mann–Whitney *U* test. Multivariable predictors for higher VAS pain scores will be determined using linear/non-linear regression. Other continuous data will be compared by the Student *t* test or Mann–Whitney *U* test for parametric and non-parametric data, as appropriate. The median and first and third quartiles will be reported for continuous variables. Categorical data will be compared by the chi-square test or Fisher’s exact test, as appropriate, with a *P* value less than 0.05 indicating significance. The 5% level will be considered significant throughout. The individual undertaking the analyses will be blinded to trial allocation.

### Data handling and dissemination of results

All patient data will be anonymised and stored on a password-protected access database. Patient records will be kept on paper in the form of the diary card questionnaires and clinical scoring sheets. These will be kept in a locked filling cabinet at each trial site.

### Criteria for electively stopping the trial or other research prematurely

The trial may be stopped prematurely due to loss of equipoise or any major adverse effect as a result of treatment in any of the treatment arms.

### Adverse events and serious adverse events

No significant adverse events are expected. An adverse event (AE) is defined as any untoward medical occurrence in a patient or clinical study subject. A serious adverse event (SAE) is any untoward and unexpected medical occurrence or effect that results in death or is life-threatening (refers to an event in which the subject was at risk of death at the time of the event). It does not refer to an event which hypothetically might have caused death if it was more severe, requires hospitalisation or prolongation of existing inpatients’ hospitalisation, results in persistent or significant disability or incapacity, or is a congenital anomaly or birth defect.

### Reporting procedures

All adverse events should be reported. Depending on the nature of the event, the reporting procedures should be adhered to; any questions concerning adverse event reporting should be directed to the Chief Investigator in the first instance.

All AEs, whether expected or not, should be recorded. In the case of SAEs, a SAE form should be completed and faxed to the Chief Investigator within 24 h. All SAEs should be reported to the Research Ethical Committee when, in the opinion of the Chief Investigator, the event was ‘related’ (i.e. resulted from the administration of any of the research procedures) and ‘unexpected’ (i.e. an event that is not listed in the protocol as an expected occurrence). Reports of related and unexpected SAEs should be submitted within 15 days of the Chief Investigator becoming aware of the event, using the NRES (National Research Ethics Service) SAE form for non-investigational medicinal product studies. Local investigators should report any SAEs as required by their Local Research Ethics Committee, Sponsor and/or Research & Development Office.

### Confidentiality

The Chief Investigator will preserve the confidentiality of participants taking part in the study and is registered under the Data Protection Act.

### Indemnity

Singapore General Hospital, Singapore holds negligent harm and non-negligent harm insurance policies which apply to this study. Patients attending Roscommon University Hospital, Ireland for their care will be eligible for compensation for negligent or non-negligent harm via the Clinical Indemnity Scheme.

## Discussion

The role of compression following endovenous interventions for superficial venous reflux remains unclear. If indicated, the optimal duration of treatment is also obscure. Non-tumescent, non-thermal (NTNT) endovenous techniques are increasingly used as they facilitate a more office-based approach to venous interventions. The COMMOCA trial will provide valuable data regarding the role of compression as an adjunct to one of the NTNT techniques. The primary aim of the study is to assess patient’s pain scores for the first 10 days post procedure using a visual analogue scale. Secondary aims include an assessment of patient compliance with compression, quality of life scores, clinical effectiveness, rates of bruising and phlebitis, time taken to return to normal activities, patient satisfaction and occlusion rate at 6 months.

This trial has some potential weaknesses, including the simple randomisation technique which has been chosen. This may lead to issues such as the production of unequal sample sizes. Additionally, the potential for losses to follow-up is a weakness in any trial of venous interventions. Short-term outcomes such as pain score are relatively straightforward to capture. However, early recurrence and recanalisation rates are of major concern to interventionists and require longer follow-up. COMMOCA is set in two established ambulatory venous centres with pathways for follow-up already in place. Between them, the two centres undertake over 1000 superficial venous procedures per annum. Six-month follow-up completion rates will be monitored closely throughout the trial. If necessary, the sample size will be recalculated to reflect a greater than expected drop-out rate and ensure sufficient statistical power regarding recanalisation rates at the end of the trial.

## Trial status

Study Protocol Version 1.4 dated 29 January 2018. Recruitment has not yet commenced.

## Supplementary information

**Additional file 1.** EQ-5D quality of life questionnaire.

**Additional file 2.** AVVQ quality of life questionnaire.

**Additional file 3.** CIVIQ quality of life questionnaire.

**Additional file 4.** CEAP classification.

**Additional file 5.** Venous Clinical Severity Score.

**Additional file 6.** Patient pain diary (visual analogue scale).

**Additional file 7.** Patient diary.

**Additional file 8.** SPIRIT Checklist: Recommended items to address in a clinical trial protocol and related documents.

## Data Availability

The datasets generated and/or analysed during the current study are not publicly available as recruitment has not yet commenced, but final data will be available from the corresponding author on reasonable request following study completion.

## Abbreviations

ABPI: Ankle–Brachial Pressure Index
AE: Adverse event
AVVQ: Aberdeen Varicose Vein Questionnaire
CEAP: Clinical–Etiological–Anatomical–Pathophysiology
CIVIQ: ChronIc Venous Insufficiency quality of life Questionnaire
COMMOCA: COMpression Therapy Following MechanO-Chemical Ablation
CVD: Chronic venous disease
EQ-5D: EuroQol 5 Dimensions (standardised instrument for use as a measure of health outcome)
EVLT: Endovenous laser therapy
GSV: Great saphenous vein
IUP: International Union of Phlebology
MOCA: Mechano-chemical ablation
NICE: National Institute for Clinical Excellence
NRES: National Research Ethics Service
RFA: Radiofrequency ablation
SAE: Serious adverse event
SPIRIT: Standard Protocol Items: Recommendations for Interval Trials
SSV: Short saphenous vein
STS: Sodium tetradecyl sulphate
VAS: Visual analogue scale
VCSS: Venous clinical severity score
