# Supplementary figures and images for: Compression therapy following ClariVein® ablation therapy: a randomised controlled trial of COMpression Therapy Following MechanO-Chemical Ablation (COMMOCA)
Source: Trials. 2019 Dec 5;20:678. doi: 10.1186/s13063-019-3787-4 (PMC6894465; doi:10.1186/s13063-019-3787-4)

**Quality of Life Questionnaire**

**
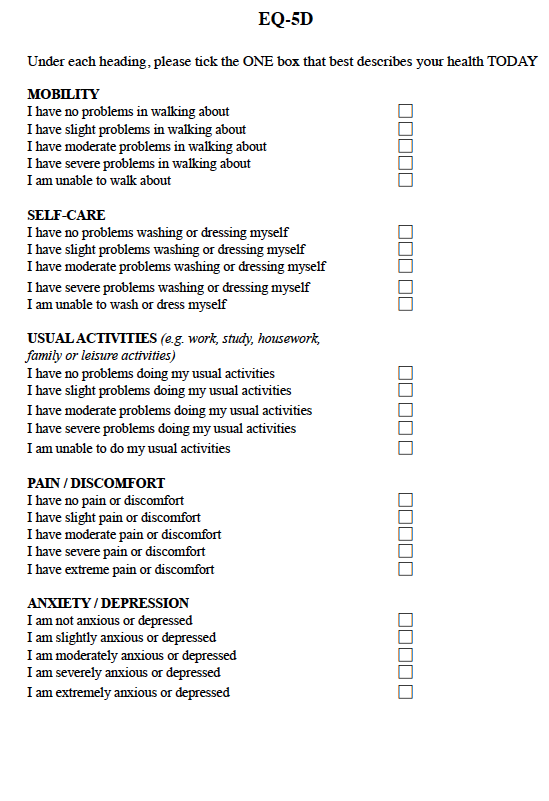
**

Supplement: Supplementary file 1 — Additional file 1. EQ-5D quality of life questionnaire. [file 13063_2019_3787_MOESM1_ESM.docx]
