# Supplementary material for: Compression therapy following ClariVein® ablation therapy: a randomised controlled trial of COMpression Therapy Following MechanO-Chemical Ablation (COMMOCA)
Source: Trials. 2019 Dec 5;20:678. doi: 10.1186/s13063-019-3787-4 (PMC6894465; doi:10.1186/s13063-019-3787-4)
Supplement: Supplementary file 2 — Additional file 2. AVVQ quality of life questionnaire. [file 13063_2019_3787_MOESM2_ESM.docx]

**Aberdeen Varicose Veins Questionnaire**

|  | **YOUR VARICOSE VEINS** |  |
| --- | --- | --- |

**1. Please draw in your varicose veins in the diagram(s) below:-**

Legs viewed Legs viewed


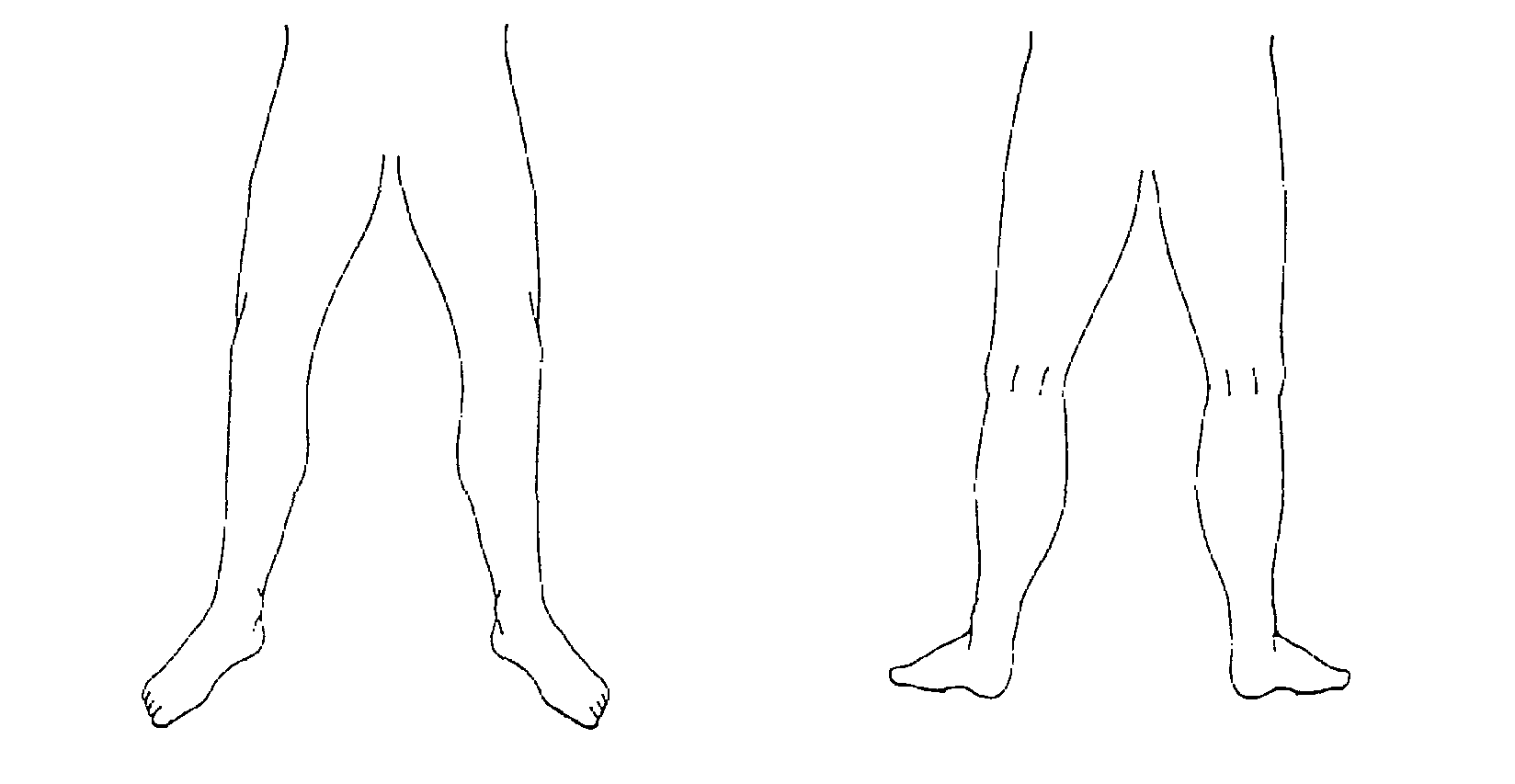
 from front from back

**2. In the last two weeks, for how many days did your varicose veins cause you pain or ache?**

| *(Please tick one box for each leg)* | R Leg | | | L Leg | | |
| --- | --- | --- | --- | --- | --- | --- |
| None at all | |  |  | |  |  |
| Between 1 and 5 days | |  |  | |  |  |
| Between 6 and 10 days | |  |  | |  |  |
| For more than 10 days | |  |  | |  |  |

**3. During the last two weeks, on how many days did you take painkilling tablets for your varicose veins?**

| *(Please tick one box for each leg)* | R Leg | | | L Leg | | |
| --- | --- | --- | --- | --- | --- | --- |
| None at all | |  |  | |  |  |
| Between 1 and 5 days | |  |  | |  |  |
| Between 6 and 10 days | |  |  | |  |  |
| For more than 10 days | |  |  | |  |  |

**4. In the last two weeks, how much ankle swelling have you had?**

| *(Please tick one box)* None at all |  |
| --- | --- |
| Slight ankle swelling |  |

| Moderate ankle swelling (eg. causing you  to sit with your feet up whenever possible) |  |
| --- | --- |

| Severe ankle swelling (eg. causing you  difficulty putting on your shoes) |  |
| --- | --- |

**5. In the last two weeks, have you worn support stockings or tights?**

| *(Please tick one box for each leg)* | R Leg | | | L Leg | | |
| --- | --- | --- | --- | --- | --- | --- |
| No | |  |  | |  |  |

| Yes, those I bought myself without  a doctor's prescription |  |  |  |
| --- | --- | --- | --- |

| Yes, those my doctor prescribed for  me which I wear occasionally |  |  |  |
| --- | --- | --- | --- |

| Yes, those my doctor prescribed for  me which I wear every day |  |  |  |
| --- | --- | --- | --- |

**6. In the last two weeks, have you had any itching in association with your varicose veins?**

| *(Please tick one box for each leg)* | R Leg | | | L Leg | | |
| --- | --- | --- | --- | --- | --- | --- |
| No | |  |  | |  |  |
| Yes, but only above the knee | |  |  | |  |  |
| Yes, but only below the knee | |  |  | |  |  |
| Both above and below the knee | |  |  | |  |  |

**7. Do you have purple discolouration caused by tiny blood vessels in the skin, in association with your varicose veins?**

| *(Please tick one box for each leg)* | R Leg | | | L Leg | | |
| --- | --- | --- | --- | --- | --- | --- |
| No | |  |  | |  |  |
| Yes | |  |  | |  |  |

**8. Do you have a rash or eczema in the area of your ankle?**

| *(Please tick one box for each leg)* | R Leg | | | L Leg | | |
| --- | --- | --- | --- | --- | --- | --- |
| No | |  |  | |  |  |

| Yes, but it does not require any treatment  from a doctor or district nurse |  |  |  |
| --- | --- | --- | --- |

| Yes, and it requires treatment from  my doctor or district nurse |  |  |  |
| --- | --- | --- | --- |

**9. Do you have a skin ulcer associated with your varicose veins?**

| *(Please tick one box for each leg)* | R Leg | | | L Leg | | |
| --- | --- | --- | --- | --- | --- | --- |
| No | |  |  | |  |  |
| Yes | |  |  | |  |  |

**10. Does the appearance of your varicose veins cause you concern?**

| *(Please tick one box)* No |  |
| --- | --- |

| Yes, their appearance causes  me slight concern |  |
| --- | --- |

| Yes, their appearance causes  me moderate concern |  |
| --- | --- |

| Yes, their appearance causes  me a great deal of concern |  |
| --- | --- |

**11. Does the appearance of your varicose veins influence your choice of clothing including tights?**

| *(Please tick one box)* No |  |
| --- | --- |
| Occasionally |  |
| Often |  |
| Always |  |

**12. During the last two weeks, have your varicose veins interfered with your work/ housework or other daily activities?**

| *(Please tick one box)* No |  |
| --- | --- |

| I have been able to work but my work  has suffered to a slight extent |  |
| --- | --- |

| I have been able to work but my work  has suffered to a moderate extent |  |
| --- | --- |

| My veins have prevented me from  working one day or more |  |
| --- | --- |

**13. During the last two weeks, have your varicose veins interfered with your leisure activities (including sport, hobbies and social life)?**

| *(Please tick one box)* No |  |
| --- | --- |

| Yes, my enjoyment has suffered  to a slight extent |  |
| --- | --- |

| Yes, my enjoyment has suffered  to a moderate extent |  |
| --- | --- |

| Yes, my veins have prevented me taking  part in any leisure activities |  |
| --- | --- |
