# Supplementary material for: Compression therapy following ClariVein® ablation therapy: a randomised controlled trial of COMpression Therapy Following MechanO-Chemical Ablation (COMMOCA)
Source: Trials. 2019 Dec 5;20:678. doi: 10.1186/s13063-019-3787-4 (PMC6894465; doi:10.1186/s13063-019-3787-4)
Supplement: Supplementary file 3 — Additional file 3. CIVIQ quality of life questionnaire. [file 13063_2019_3787_MOESM3_ESM.docx]

**C I V I Q-14**

**SELF-QUESTIONNAIRE PATIENTS**

***In English language for UK***

Many people complain of leg pain. We would like to find out how often these leg problems occur and to what extent they affect the everyday lives of those who suffer from them.

Below you will find a list of symptoms, sensations or types of discomfort that you may be experiencing and which may make everyday life hard to bear to a greater or lesser extent. **For each symptom, sensation, or type of discomfort listed, we would like you to answer in the following way:**

Please indicate if you have experienced what is described in each sentence, and if the answer is ‘yes’, how **intense** it was. There are five possible answers, and we would like you to circle the one which best describes your situation.

Circle 1 if you feel the symptom, sensation of discomfort described does not apply to you

Circle 2, 3, 4 or 5 if you have felt it to a greater or lesser extent

**QUALITY OF LIFE WITH VENOUS INSUFFICIENCY**

| **1)**  During the past four weeks, have you had any **pain** in your **ankles** or **legs**, and how severe has this pain been?  *Circle the number that applies to you.* | | | | |
| --- | --- | --- | --- | --- |
| No  pain | Slight  pain | Moderate  pain | Considerable  pain | Severe  pain |
|  |  |  |  |  |
| 1 | 2 | 3 | 4 | 5 |
|  |  |  |  |  |

| **2)**  During the past four weeks, how much trouble have you experienced at **work** or during your **usual daily activities because of your leg problems**?  *Circle the number that applies to you.* | | | | |
| --- | --- | --- | --- | --- |
| No trouble | Slight trouble | Moderate trouble | Considerable trouble | Severe trouble |
|  |  |  |  |  |
| 1 | 2 | 3 | 4 | 5 |
|  |  |  |  |  |

| **3)**  During the past four weeks, have you **slept** **badly** because of your leg problems, and how often?  *Circle the number that applies to you.* | | | | |
| --- | --- | --- | --- | --- |
| Never | Rarely | Fairly  often | Very  often | Every  night |
| 1 | 2 | 3 | 4 | 5 |
|  |  |  |  |  |

| During the past four weeks, how much **trouble** have you experienced **carrying out the actions and activities** listed below b**ecause of your leg problems**?  For each statement in the table below, indicate how much trouble you have experiened by circling the number chosen. | | | | |  |
| --- | --- | --- | --- | --- | --- |
|  | No trouble | Slight trouble | Moderate trouble | Considerable trouble | Could not  do it |
| **4)**  Climbing several flights of stairs | 1 | 2 | 3 | 4 | 5 |
| **5)**  Crouching,  Kneeling down | 1 | 2 | 3 | 4 | 5 |
| **6)**  Walking at a brisk pace | 1 | 2 | 3 | 4 | 5 |
| **7)**  Going out for the evening, going to a wedding, a party, a cocktail party… | 1 | 2 | 3 | 4 | 5 |
| **8)**  Playing a sport, exerting yourself physically | 1 | 2 | 3 | 4 | 5 |

| Leg problems can also affect your mood. How closely do the following statements correspond to what you have felt during the past four weeks?  *For each statement in the table below, circle the number that applies to you.* | | | | |  |
| --- | --- | --- | --- | --- | --- |
|  | Not at all | A little | Moderately | A lot | Completely |
| **9)**  I have felt nervous/tense | 1 | 2 | 3 | 4 | 5 |
| **10)**  I have felt I am a burden | 1 | 2 | 3 | 4 | 5 |
| **11)**  I have felt embarrassed about showing my legs | 1 | 2 | 3 | 4 | 5 |
| **12)**  I have become irritated easily | 1 | 2 | 3 | 4 | 5 |
| **13)**  I have felt as if I am handicapped | 1 | 2 | 3 | 4 | 5 |
| **14)**  I have not felt like going out | 1 | 2 | 3 | 4 | 5 |
