# Supplementary material for: Compression therapy following ClariVein® ablation therapy: a randomised controlled trial of COMpression Therapy Following MechanO-Chemical Ablation (COMMOCA)
Source: Trials. 2019 Dec 5;20:678. doi: 10.1186/s13063-019-3787-4 (PMC6894465; doi:10.1186/s13063-019-3787-4)
Supplement: Supplementary file 4 — Additional file 4. CEAP classification. [file 13063_2019_3787_MOESM4_ESM.docx]

**Clinical-Etiology-Anatomy-Pathophysiology (CEAP Classification)^1^**

| **Clinical** | 0 | 1A | 1S | 2A | 2S | 3A | 3S | 4aA | 4aS | 4bA | 4bS | 5A | 5S | 6 |
| --- | --- | --- | --- | --- | --- | --- | --- | --- | --- | --- | --- | --- | --- | --- |
| **Etiology** | Congenital | | | Primary | | | Secondary | | | No venous cause identified | | | | |
| **Anatomy** | Superficial | | | Deep | | | Perforating | | | No venous location identified | | | | |
| **Pathology** | Reflux | | | Obstruction | | | Both | | | No venous pathology identified | | | | |

Class 0 No visible or palpable veins

Class 1 Telangiectasia, reticular veins Class 5 Skin changes and healed ulceration

Class 2 Varicose Veins Class 6 Active venous ulcers

Class 3 Oedema without skin changes

Class 4 Skin changes ascribed to venous disease A= Asymptomatic

4a) Pigmentation or eczema S= Symptomatic

4b) Lipodermatoscerosis or atrophie blanche

1. Porter JM, Moneta GL. Reporting standards in venous disease: an update. International Consensu Committee on Chronic Venous Disease. J Vasc Surg 1995;21:635-45
