# Supplementary material for: Compression therapy following ClariVein® ablation therapy: a randomised controlled trial of COMpression Therapy Following MechanO-Chemical Ablation (COMMOCA)
Source: Trials. 2019 Dec 5;20:678. doi: 10.1186/s13063-019-3787-4 (PMC6894465; doi:10.1186/s13063-019-3787-4)
Supplement: Supplementary file 5 — Additional file 5. Venous Clinical Severity Score. [file 13063_2019_3787_MOESM5_ESM.docx]

**Venous Clinical Severity Score (VCSS)**

**Please indicate right or left leg or bilateral (R, L or B)**

|  | Absent | Mild | Moderate | Severe |
| --- | --- | --- | --- | --- |
| Pain | None | Occasional, non/ no analgesia restricting | With moderate activity, occasional analgesia | Daily, severe limitations, regular analgesia |
| Varicose veins>4mm | None | Few | Multiple GSV | Extensive GSV and LSV |
| Venous oedema | None | Evening/ankle | Afternoon/ above knee | Morning/requiring elevation |
| Skin pigmentation | None | Limited and old/brown | Diffuse lower third/ purple | Wide/ purple |
| Inflammation | None | Mild cellulitis in marginal area | Moderate involving most of gaiter area | Severe cellulitis or significant eczema |
| Induration | None | Focal <5cm | Medial or lateral less than lower 1/3 | 1/3 of lower leg or more |
| Number of active ulcers | 0 | 1 | 2 | 3 |
| Active ulcer duration | None | <3 months | >3 months  <12 months | >12 months |
| Active ulcer diameter( cm) | None | <2 | 2-6 | >6 |
| Compression | Not used or non compliant | Intermittent use | Stockings worn most days | Stockings worn daily |
| Total |  |  |  |  |
