# Supplementary material for: Compression therapy following ClariVein® ablation therapy: a randomised controlled trial of COMpression Therapy Following MechanO-Chemical Ablation (COMMOCA)
Source: Trials. 2019 Dec 5;20:678. doi: 10.1186/s13063-019-3787-4 (PMC6894465; doi:10.1186/s13063-019-3787-4)
Supplement: Supplementary file 6 — Additional file 6. Patient pain diary (visual analogue scale). [file 13063_2019_3787_MOESM6_ESM.docx]

**Patient Pain Diary (VAS)**

Please put a mark on the line to indicate your maximum pain score on each day. Please also write a score from 0 to 10 for your maximum pain on each day.

***Example****: If your pain score on day 4 is roughly about 5 over 10 (10 being the worst pain imaginable), you might want to indicate it as below:*

| **Day 4** | No pain |  | 5  *\|* |  |  | Worst pain imaginable |
| --- | --- | --- | --- | --- | --- | --- |
|  |  | 0 |  |  | 10 |  |

**Study Diary:**

(0 = no pain and 10 = worst pain imaginable)

| **Day 0** | No pain | |  |  |  |  | Worst pain imaginable |
| --- | --- | --- | --- | --- | --- | --- | --- |
|  |  | | 0 |  |  | 10 |  |
| **Day 1** | No pain | |  |  |  |  | Worst pain imaginable |
|  |  | | 0 |  |  | 10 |  |
| **Day 2** | No pain | |  |  |  |  | Worst pain imaginable |
|  |  | | 0 |  |  | 10 |  |
| **Day 3** | No pain | |  |  |  |  | Worst pain imaginable |
|  |  | | 0 |  |  | 10 |  |
| **Day 4** | No pain | |  |  |  |  | Worst pain imaginable |
|  |  | | 0 |  |  | 10 |  |
| **Day 5** | No pain | |  |  |  |  | Worst pain imaginable |
|  |  | | 0 |  |  | 10 |  |
| **Day 6** | No pain | |  |  |  |  | Worst pain imaginable |
|  |  | | 0 |  |  | 10 |  |
| **Day 7** | No pain | |  |  |  |  | Worst pain imaginable |
|  |  | | 0 |  |  | 10 |  |
| **Day 8** | No pain | |  |  |  |  | Worst pain imaginable |
|  |  | | 0 |  |  | 10 |  |
| **Day 9** | No pain | |  |  |  |  | Worst pain imaginable |
|  |  | 0 | |  |  | 10 |  |
| **Day 10** | No pain |  | |  |  |  | Worst pain imaginable |
|  |  | 0 | |  |  | 10 |  |
