# Supplementary material for: Compression therapy following ClariVein® ablation therapy: a randomised controlled trial of COMpression Therapy Following MechanO-Chemical Ablation (COMMOCA)
Source: Trials. 2019 Dec 5;20:678. doi: 10.1186/s13063-019-3787-4 (PMC6894465; doi:10.1186/s13063-019-3787-4)
Supplement: Supplementary file 7 — Additional file 7. Patient diary. [file 13063_2019_3787_MOESM7_ESM.docx]

**Patient Diary**

**Please indicate at what stage you were able to return to work and your normal daily activities (the activities you were able to do prior to treatment).**

**Please also indicate the day when you stopped wearing the compression stockings (if provided).**

**(Please tick one box)**

|  | **Day I was able to resume my normal activities** | **Day I returned to work** | **Day I stopped wearing compression stockings (if provided)** |
| --- | --- | --- | --- |
| **Day of surgery** |  |  |  |
| **Day after surgery** |  |  |  |
| **2 days after surgery** |  |  |  |
| **3 days after surgery** |  |  |  |
| **4 days after surgery** |  |  |  |
| **5 days after surgery** |  |  |  |
| **6 days after surgery** |  |  |  |
| **7 days after surgery** |  |  |  |
| **8 days after surgery** |  |  |  |
| **9 days after surgery** |  |  |  |
| **10 days after surgery** |  |  |  |
| **>10 days after surgery** |  |  |  |

Please return to:

Tjun Tang MD FRCS

Consultant Vascular & Endovascular Surgeon

Adjunct Assistant Professor (YLL-NUS)

Department of General Surgery

Changi General Hospital

2 Simei Street 3

Singapore 529889
